# Outcome of complex non-unions of femoral fractures managed with Ilizarov method of distraction osteogenesis

**DOI:** 10.12669/pjms.35.4.244

**Published:** 2019

**Authors:** Karim Bakhsh, Faridullah Khan Zimri, Atiq-Ur-Rehman, Eid Mohammad, Muhammad Saaiq

**Affiliations:** 1 Dr. Karim Bakhsh, FCPS. Department of Orthopedics, Civil Hospital, Bolan Medical College, Quetta, Pakistan; 2 Dr. Faridullah Khan Zimri, FCPS. Department of Orthopedics, NIRM, Islamabad, Pakistan; 3 Dr. Atiq-Ur-Rehman, FCPS. Department of Orthopedics, Civil Hospital, Bolan Medical College, Quetta, Pakistan; 4 Dr. Eid Mohammad FCPS. Department of Orthopedics, Civil Hospital, Bolan Medical College, Quetta, Pakistan; 5 Dr. Muhammad Saaiq, FCPS. Department of Orthopedics, NIRM, Islamabad, Pakistan

**Keywords:** Ilizarov technique, Ilizarov method, Non unions of femur fractures

## Abstract

**Objective:**

To evaluate the management outcome of complex non-union of femoral fractures with Ilizarov method in terms of bone union, functional results and any complications.

**Methods:**

This case series study was carried out at the Departments of Orthopedic Surgery, National Institute of Rehabilitation Medicine (NIRM), Islamabad and Civil hospital, Quetta over a period of three and half years, January 1, 2015 to June 30,2018.

**Results:**

There were 50 patients in the study. There were 48(96%) males and 2(4%) females. The ages ranged between 17-54 years with a mean of 33.58±8.9 years. As per ASAMI criteria, the bone results were excellent in 17(34%), good in 30(60%), fair in 1(2%) and poor in 2(4%) patients. The functional results were excellent in 15(30%), good in 24(48%), fair in 8(16%) and poor in 3(6%). The bone union rate was 98% whereas infection eradication rate was 94%. The most frequent complications were pin tract infection affecting 80% patients, knee stiffness 60% patients and K-wires loosening 20% patients.

**Conclusion:**

The Ilizarov method provides an effective solution to address the complex non-union of femur fractures. It helps to ensure fracture healing, eradicates infection and provides good functional outcome. The attended complications are mild to moderate and manageable with conservative means.

## INTRODUCTION

Lower limb trauma and the associated skeletal injuries constitute a major workload of the orthopedic surgeons in developing countries like Pakistan. Fracture non-union of femur is one formidable issue which may be encountered in a significant proportion of these patients. Fractures may fail to progress to biological union owing to a host of underlying reasons. For instance, severe injuries with reduced arterial flow, associated soft tissue trauma, failure to ensure appropriate initial fracture management and osteomylitis. This may be further complicated by additional factors like bone loss, deformity and leg length discrepancy.^1^-^3^

We employed Ilizarov method for managing non union of femur. This method is based on the fundamental principle of ‘tension stress’. It involves gradual traction or stress on living tissues that induces regeneration and active growth of body tissues such as the bone, muscle, associated skin and soft tissues. The Ilizarov method has emerged as the gold standard for addressing the non union as well as associated bone loss, deformity and leg length discrepancy.^3^,^4^

The present study was carried out to document the management outcome of complex non unions of femur fractures managed with Ilizarov method in terms of bone results, functional outcome and any complications encountered during the course of treatment.

## METHODS

We undertook this prospective descriptive case series study at the Departments of Orthopedic Surgery, National Institute of Rehabilitation Medicine (NIRM), Islamabad and Civil hospital, Bolan Medical College, Quetta over a period of three and half years from Jan 01, 2015 to June 30, 2018. The study protocol was approved by the ethics committees of the hospitals. Written informed consent was taken from the patients.

The study included all patients whose complex non unions of femoral fractures were managed with Ilizarov method. We defined complex non unions of femoral fractures as an established non-union (of at least 6-months duration) with one or more of the following criteria: a) infection at the site of non-union, b) a bone defect > 4 cm and c) a previous failed attempt to achieve union with at least one supplementary intervention such as bone grafting or exchange nailing. Our exclusion criteria included patients not willing for prolonged treatment with Ilizarov method.

All the patients were hospitalized for management. They were initially evaluated with history, physical examination and baseline investigations. Standard X-rays and tissue for culture sensitivity of the affected bone were performed. Standard wound care and antibiotic therapy was instituted according to the culture sensitivity reports of the wound specimens.

The surgeries were performed under spinal or general anesthesia. The patient was positioned supine on a radiolucent traction operating table. The ilizarov external fixator was applied in a standard fashion. We employed hybrid technique using combination of half pins and K-wires.

A bifocal compression distraction technique (compression of the non-union with distraction at the corticotomy) was employed in non-unions where the bone gap was > 2cm. Monofocal treatment (simple stabilization of the non-union and compression) was used in cases of non-unions where the gap was > 1-2cm. A standard bifocal circular frame for complex non-union of the femur consisted of 3-4 rings and proximal semicircular ring and arches. One week latency period was allowed before starting distraction. The rate of distraction was 1mm per day, performed as 0.25mm every six hourly. The ilizarov frame was retained until final union was achieved. Our definition of union was the presence of bridging trabeculae on 3 cortices, absence of pain on dynamization and the absence of movement at the union site when screened using fluoroscopy.

The Association for the Study and Application of Methods of Ilizarov (ASAMI) criteria was used to evaluate the bone results and functional results in our study. Various other outcome measurements included bone results, functional results and any complications encountered through the course of treatment.

We employed SPSS version 17 (SPSS Inc, Chicago, Illinois, USA) to carry out statistical analysis. Various descriptive statistics were used to calculate the outcome measures of interest.

## RESULTS

There were 50 patients in the study, 48(96%) males and 2(4%) females, ages ranged between 17-54 years with a mean of 33.58±8.9 years.The various bacteria cultured from the infected bones included Staphylococcus aureus 40(80%), Pseudomonas aeruginosa 17(34%), Methicillin Resistant Staphylococcus aureus 8(16%), Escherichia coli 8(16%) and various other bacterial species 7(14%). Twenty one wound cultures were positive for polymicrobials.

The time interval between the primary injury the definitive management with the Ilizarov method ranged from 10 to 20 months with a mean of 11 months. It was 9 months among 7(14%) patients, 10 months among 15(30%) patients, 11 months among 19(38%) patients, 12 months among 7(14%) patients and 20 months among 2(4%) patients. At the time of presentation for definitive management with Ilizarov method, the surgical interventions already undergone by the patients included three surgeries among 27 (54%)patients, two surgeries among 11(22%) patients, four surgeries among 9(18%) patients and one surgery among three (6%) patients. The average number of surgeries were 2.84 per patient. Areas of the femur involved in the gap non union included distal femur(n=31), mid femur(n=17) and proximal femur(n=2).

At the time of application of Ilizarov fixator, all patients had severe limitation of range of motion of the knee, with a mean ROM of 50° (range, 0°-70°). The mean ROM at the time of frame removal was 40° (range, 20°-90°). The mean ROM at 6 months following aggressive physiotherapy was 90° (range, 80°-110°).

Preoperatively the skeletal defects or bone gaps ranged from 1-7 cm with a mean of 3.62 cm. It was 1cm in 5(10%) patients, 2cm in 7(14%) patients, 3cm in 9(18%) patients, 4 cm in 16(32%) patients, 5 cm in 9(18%) patients, 6 cm in 2(4%) patients and 7 cm in 2 (4%) patients. Postoperatively at the time of removal of frame, only 9(18%) patients had limb shortening of over 2.5 cm. The mean shortening among them was 3.1cm (range 3-4 cm). Among all these cases further treatment was deliberately curtailed in order to reduce the duration of this complex treatment.

According to ASAMI criteria, bone results are displayed in Table-I whereas the functional results are shown in Table-II. Bone union was achieved among all except two patients. (98% bone union rate). Infected was eradicated among 47(94%) patients. The bone transport time ranged from 29-135 days with a mean of 65 days.

**Table I:** Bone evaluation results among the patients. (n=50).

	Bone evaluation	Guidelines parameters	Number/ Percentage
1	Excellent	Eradication of the infection, bone union, deformities <7° and LLD <2.5 cm	17(34%)
2	Good	Bone union + any two of the following items eradication of infection, <7° deformity LLD <2.5 cm	30(60%)
3	Fair	Bone union, persistence of infection, less than 7° deformities and more than 2.5 cm limb length discrepancies	1(2%)
4	Poor	Bone nonunion + infection + deformity >7° + LLD >2.5 cm	2(4%)

**LLD:** Limb length discrepancy

**Table II:** Functional results among the patients. (n=50).

	Functional evaluation	Guidelines parameters	Number/ Percentage
1	Excellent	Active, no limp, minimal knee stiffness (loss of <15° knee extension), no RDS, and significant pain	15(30%)
2	Good	Active, no limp, pain significant, no RDS and 20° loss of knee extension	24(48%)
3	Fair	Active, limping, knee stiffness, RSD and significant pain	8(16%)
4	Poor	Inactive and unable to return to daily activities	3(6%)

**RSD:** Reflex sympathetic dystrophy

The complications encountered included pin tract infection among 40(80%) patients, knee stiffness among 30(60%) patients, K-wires loosening in 10(20%) patients, delayed consolidation in 5(10%) patients, axial deviation during bone transport in 3(6%) patients, skin invagination requiring plastic surgical correction in 4(8%) patients, persistent non union in 2(4%) patients and femoral re fracture in 1(2%) patient. There was no mortality.

## DISCUSSION

The Ilizarov method has stood the test of time for several decades in addressing a host of skeletal injuries and deformities. The technique was pioneered by the Russian inventor Ilizarov in 1951. The technique involves passing percutaneous wires, tensioning them adequately and attaching them to the rings of the circular metal frame. With this rigid arrangement, it is possible to simultaneously obtain fracture compression, bone distraction histiogenesis, bone distraction, bone lengthening and deformity correction. The fixator is strong enough to allow ambulation and immediate weight bearing.^1^-^4^

Prior to the development of the Ilizarov method, the treatment of bone loss and non-unions caused by traumatic injuries or infections involved the use of a variety of other surgical techniques such as the use of cancellous autogenous bone grafts, vascularized bone grafts, structural allografts and artificial bone substitutes, employment of internal fixation devices. The treatment of non-unions using the Ilizarov method depends on the type of nonunion, namely hypertrophic and atrophic. Since the hypertrophic non-unions have sufficient vascularity to promote bone healing but lack the necessary structural stability, Ilizarov recommended gradual compression of the two surfaces of the nonunion to promote consolidation, followed by gradual distraction to compensate for the associated bone loss. In case of atrophic non-unions, there is significantly reduced bone healing potential, compression and distraction are carried out simultaneously at different locations in the affected bone. Generally speaking, the bone is compressed at the site of the nonunion and a separate osteotomy is performed at another part of bone fragment, and distraction is performed there. The two goals of treatment are achievement of fracture union and lengthening. These two can be split into two interventions sequentially. First surgery may be aimed to achieve union. The second intervention may be later instituted to correct the leg length discrepancy and any associated deformities.^5^-^8^

In the majority of our patients the non union was associated with infection. We undertook an initial thorough debridement of the devitalized bone before instituting the distraction osteogenesis through Ilizarov method. Following the bone debridement a bone gap is created. The combination of the bone gap and associated infection precludes the potential use of an internal fixator. Hence we need an external fixation device. The Ilizarov method offers the most prudent option to address these issues as it on one hand provides stable fixation which is mechanically strong enough to allow mobilization and on other hand ensures new bone formation, lengthening and deformity correction. The technique helps to eliminate infection by increasing the local blood supply and vascularity of the osteomyelitic focus. Hence the famous notion “osteomyelitis burns in the fire of regeneration”. Our favorable results with Ilizarov method for infected nonunion of the femur conform to several published studies.^9^-^12^

In this study, we used the ASAMI criteria for bone evaluation and measuring the functional outcome. This system incorporates four main criteria for evaluating the bone results. These include bone/ fracture union, infection eradication, deformity correction and limb length discrepancy (LLD). The functional evaluation is based on five criteria. These include limp, joint stiffness, reflex sympathetic dystrophy, pain and inactivity.^13^

In our study the bone results were excellent in 34% and good in 60% patients. Our outcome results conform favorably to most of the published literature.^8^,^14^-^18^ We had infection eradication among 94% patients. Krishnan A et al.^12^ reported elimination of infection among nineteen of their twenty patients with infected non union of femur who were managed with the Ilizarov method.

In this study the functional results were excellent in 30% whereas good in 48% patients. Our outcome results conform favorably to the published studies.^8^,^14^-^17^ Krishnan A et al.^12^ 85% and 60% of patients had good-to-excellent bone and functional results, respectively.^5^-^8^

In this study, the major share of complications was constituted by pin tract infection, knee stiffness and K-wires loosening. Krishnan A et al.^12^ reported re-fracture, femoral bowing of 20° and shortening of 2.5 cm, one each in their series. Palatnik Y^4^ reported. pain during the distraction phase, pin-tract infections and decreased range knee motion as their most common complications. Our findings conform to most of the published literature.^4^,^8^,^19^ Complications are intrinsic to the Ilizarov method however their frequency and severity decrease with increasing experience of the surgical team as well as improvement in the physiotherapy back up facility.

The Ilizarov method of treatment is attended by a host of challenges through the course of treatment. Firstly, it a relatively prolonged treatment and we initially counsel the patients specifically regarding the lengthy period of the fixator application. Also several complications are subsequently encountered through the course of treatment. For instance, pin tract infections, loosening of K-wires, and knee stiffness. These issues often lead to compliance issues on part of the patients. These factors at times lead to deliberate shortening of the treatment and discontinuation before achievement of the desired leg length.^4^-^8^,^16^-^21^

Physiotherapy is an important adjunct to any good orthopedic surgical facility. Elsewhere in the world, physiotherapy has become an integral part of orthopedic surgery units. Unfortunately the process of integration of physiotherapy with orthopedic surgery is still in infancy in our country. Additionally compliance with home physiotherapy regimens is also very poor amongst our patients. Owing to these factors, we have higher rate of initial knee stiffness among our patients.

Most of the previously published local studies and literature on Ilizarov method have focused on the management of tibial fractures and correction of congenital deformities.^18^,^20^-^22^ Hence there is relative scarcity of similar local studies pertaining to the management of complex femoral non unions. Given the context of femoral fractures, the application of Ilizarov method is more demanding for the patient as well as the surgeon. The bulky muscular surroundings of the femur renders it a difficult bone to treat with this method. Transfixation of the local tissues is more frequently associated with pain and discomfort through the course of treatment. Additionally there is more frequent problem of knee stiffness and higher frequency of pin track problems. Our study should prompt future local multicenter studies to confirm and improve upon our study. This will help to generate local evidence base for better management of patients with complex non unions of femoral fractures.

## CONCLUSION

The Ilizarov method provides an effective solution to address the complex non-union of femur fractures. It helps to ensure fracture healing, eradicates infection and provides good functional outcome. The attended complications are mild to moderate and manageable with conservative means.

### Authors’ Contributions

**FUKZ, KB, AUR & MS**: designed the study and prepared the manuscript.

**MS, EM & AUR**: Performed data collection and analyzed the results.

All authors approved the manuscript.
